# Propagation of Ultrasonic Guided Waves in Composite Multi-Wire Ropes

**DOI:** 10.3390/ma9060451

**Published:** 2016-06-06

**Authors:** Renaldas Raisutis, Rymantas Kazys, Liudas Mazeika, Vykintas Samaitis, Egidijus Zukauskas

**Affiliations:** Ultrasound Institute of Kaunas University of Technology, K. Barsausko 59, Kaunas Lt-51423, Lithuania; rymantas.kazys@ktu.lt (R.K.); liudas.mazeika@ktu.lt (L.M.); vykintas.samaitis@ktu.lt (V.S.); e.zukauskas@ktu.lt (E.Z.)

**Keywords:** multi-wire ropes, modelling, ultrasonic guided waves, dispersion curves, 43.35.Yb, 43.35.Zc, 43.60.Qv

## Abstract

Multi-wire ropes are widely used as load-carrying constructional elements in bridges, cranes, elevators, *etc*. Structural integrity of such ropes can be inspected by using non-destructive ultrasonic techniques. The objective of this work was to investigate propagation of ultrasonic guided waves (UGW) along composite multi-wire ropes in the cases of various types of acoustic contacts between neighboring wires and the plastic core. The modes of UGW propagating along the multi-wire ropes were identified using modelling, the dispersion curves were calculated using analytical and semi-analytical finite element (SAFE) techniques. In order to investigate the effects of UGW propagation, the two types of the acoustic contact between neighboring wires were simulated using the 3D finite element method (FE) as well. The key question of investigation was estimation of the actual boundary conditions between neighboring wires (solid or slip) and the real depth of penetration of UGW into the overall cross-section of the rope. Therefore, in order to verify the results of FE modelling, the guided wave penetration into strands of multi-wire rope was investigated experimentally. The performed modelling and experimental investigation enabled us to select optimal parameters of UGW to be used for non-destructive testing.

## 1. Introduction

Multi-wire ropes are widely used in different sectors of industry as load-carrying constructional elements in bridges, cranes, elevators, *etc*. Due to environmental and operational conditions and fatigue caused by rotational stresses, multiple defects may develop in the internal structure of multi-wire ropes. One of the promising methods for investigation of non-homogeneities of the internal structure of wire ropes can be application of ultrasonic guided waves (UGW) [1,2,3,4,5,6,7,8,9,10]. UGW are suitable for detection of various structural non-homogeneities, because they can propagate long distances and are quite sensitive both to surface and internal defects [11,12,13]. However, in the case of multi-wire ropes, the key issues are whether UGW will propagate only in one particular wire/strand, in which it had been excited, or in the whole composite multi-wire rope, and how the penetration of UGW will depend on the excitation method and the type of a mechanical contact between neighboring wires.

The objective of this work was to investigate the propagation of ultrasonic guided waves (UGW) along composite multi-wire ropes in the cases of various types of acoustic contacts between neighboring wires and the plastic core.

## 2. The Object of Investigation

A multi-wire steel rope to be investigated using ultrasound by itself is a complex composite structure in which multiple reflections, scattering, and the presence of multiple wave modes of UGW occur. Therefore, in order to understand the effects of UGW propagating along the multi-wire rope and penetration into the internal structure, determination of type of the acoustic contact between neighboring wires is necessary. In addition, the optimal parameters of UGW used for non-destructive testing of the particular objects are necessary to be investigated and selected.

**Figure 1:**
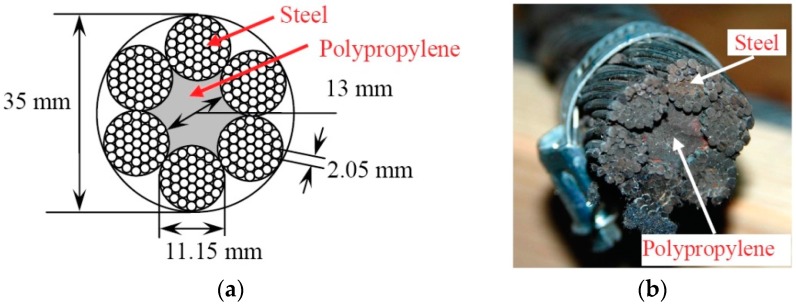
The rope under investigation: (**a**) schematic view; and (**b**) photo of the cross-section of the multi-bundle and multi-wire twisted rope.

As an object of investigation, a multi-wire steel rope consisting of six strands, each with the diameter of 11.15 mm, had been selected. The core of the multi-wire rope had been filled with polypropylene, and each strand consisted of a bundle of approximately 31 wires, each with the diameter of 2.05 mm. The cross-sectional view of the investigated composite multi-wire rope is presented in Figure 1. In order to simplify further analysis the assumption was made that the structure of rope consists of straight strands.

### 2.1. Dispersion Curves of Propagating Guided Waves

Dispersion curves of propagating UGW along the object under investigation show the distribution of phase or group velocities in a particular frequency bandwidth. Therefore, in order to identify the particular modes of UGW, which propagate along the object under investigation and select suitable ones for non-destructive testing, the dispersion curves of phase and group velocities must be calculated. There are several techniques for calculation of the dispersion curves of UGW: semi-analytical finite element (SAFE) technique [14,15,16,17,18,19,20] and analytical ones [21]. The SAFE technique is more attractive for analysis of the objects with an arbitrary geometry of the cross-section (e.g., rails, bars, *etc.*). The SAFE technique has been used by Treyssede and Laguerre for investigation of UGW propagation along multi-wire helical strands with a metal cylindrical core [4,7,9,10,14]. The wires were assumed to be solidly connected [4,9,14]. They have found that the wave numbers and energy velocities of axisymmetric modes, such as *L*(0,1) and *T*(0,1), also flexural mode *F*(1,1) at low frequencies and small helix lay angles (less than 15 degrees) of helical waveguides are very similar to the straight (not twisted) and twisted (helical) wires of multi-wire helical waveguides [4,9,14]. However, the cut-off region of the *L*(0,1)-like mode was observed at a notch frequency [4,9,14]. The advantage of the analytical technique, comparing it to SAFE, is a possibility to determine the frequency dependent guided wave leakage losses in the case of submerged wire ropes, for instance, as in cement grout or rock-embedded wire ropes [20,21].

**Figure 2:**
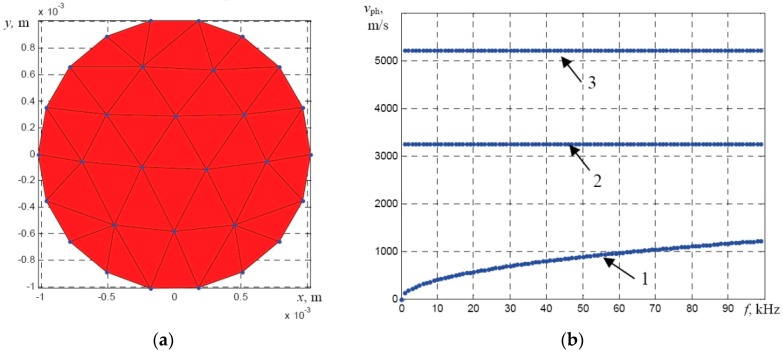
The mesh of the SAFE model of a single wire with diameter of 2.05 mm (**a**) and the dispersion curves of the phase velocity of guided wave modes (**b**): 1—flexural *F*(1,1) mode; 2—torsional *T*(0,1) mode; 3—longitudinal *L*(0,1) mode.

In order to calculate dispersion curves for a multi-wire rope with a polymer core, several tasks are necessary to be solved. At first, the stranding of wires was eliminated and calculation of dispersion curves was divided into several stages. During the first stage, the dispersion curves of UGW were calculated using the SAFE technique for a single wire having the diameter of 2.05 mm (Figure 2a,b). The material properties used for the SAFE calculations of the dispersion curves are summarized in Table 1.

During the SAFE calculations the triangular finite elements were used in order to obtain the desired accuracy, the average spatial size of element was set to 1 mm, which corresponds to 15 elements per wavelength, according to the minimum wavelength of the slowest *F*(1,1) mode at 100 kHz frequency.

Calculation results of UGW propagation along a single wire having the diameter of 2.05 mm (Figure 2a) demonstrated that only fundamental modes propagate in the frequency band up to 100 kHz (Figure 2b). The dispersion curves also show that, in the selected frequency region, the fastest mode is longitudinal *L*(0,1) and the slowest mode—flexural *F*(1,1), whereas the torsional mode *T*(0,1) is not dispersive.

**Figure 3:**
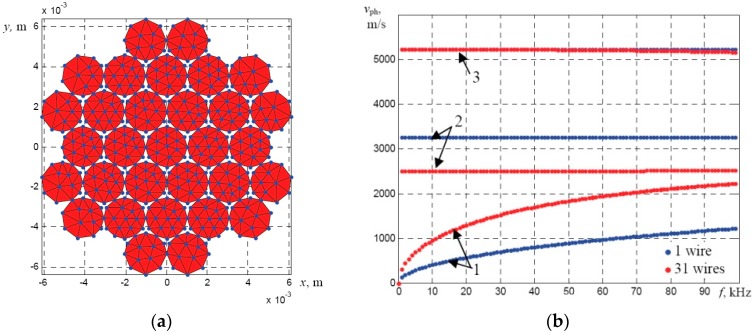
The mesh of the SAFE model of a bundle of solidly connected 31 wires (overall diameter 11.15 mm) (**a**) and comparison of the phase velocity dispersion curves of the guided wave modes (**b**) propagating along: one wire (**blue**), bundle of 31 wires (**red**): 1—flexural-like *F*(1,1) mode; 2—torsional-like *T*(0,1) mode; 3—axial-like *L*(0,1) mode.

The obtained results demonstrated that in this frequency range only fundamental modes propagate in a single wire. Typically, a multi-wire rope structure is more complicated, so, during the second stage the dispersion curves of the strand with 31 wires (the diameter of each wire 2.05 mm) having the overall diameter of 11.15 mm were calculated. In this case it was assumed that there is a solid acoustic contact between neighboring wires at their contact points. The cross-section of the meshed strand of 31 wires (the overall diameter 11.15 mm) is presented in Figure 3a. The obtained phase velocity dispersion curves and the comparison with the dispersion curves of the single wire (diameter 2.05 mm) are presented in Figure 3b. It should be noted that in the case of a bundle of wires or multi-wire rope the modes should be named as the axial-like *L*(0,1), the flexural-like *F*(1,1), and the torsional-like *T*(0,1) modes.

**Figure 4:**
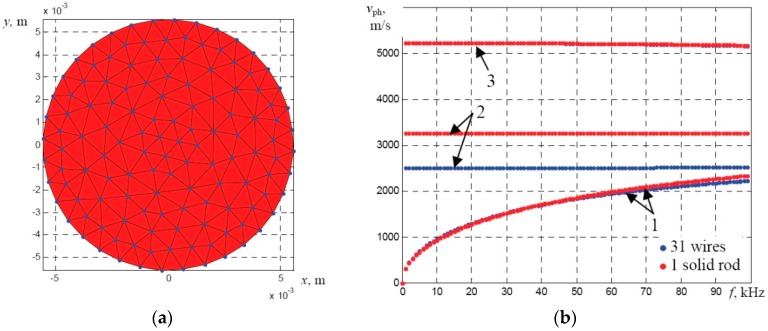
The mesh of the SAFE model of a strand that assumed to be equivalent to the bundle of solidly connected 31 wires having the overall diameter of 11.15 mm (**a**) and comparison of the phase velocity dispersion curves of the guided wave modes (**b**) propagating along the bundle of 31 wires (**blue**) and the solid rod of same diameter (**red**): 1—flexural-like *F*(1,1) mode; 2—torsional-like *T*(0,1) mode; 3—axial-like *L*(0,1) mode.

However, the numerical analysis of the multi-wire rope with the six strands consisting of 31 wires each and the polymer core is quite complicated due to limited computational resources of SAFE and the expected memory overflow. Therefore, the assumption was made that each strand can be replaced by a solid rod, having the same diameter (11.15 mm). The comparison of the dispersion curves of the UGW propagating in a multi-wire strand of 31 wires and in the solid rod are presented in Figure 4a,b. Comparison of these two cases demonstrate that the phase velocities of the *F*(1,1) and *L*(0,1) modes are similar and the velocity of the *T*(0,1) mode in the strand of 31 wires is reduced by 23% in comparison to the phase velocities in the case of a solid rod. The calculation results demonstrate that a solid circular rod having the diameter of 11.15 mm is not fully equivalent to the meshed bundle of 31 wires but this replacement can be done for analysis of fundamental modes of UGW (especially *L*(0,1) and *F*(1,1)) at frequencies below 100 kHz.

This comparative analysis was made in order to be sure that replacement of the bundle of solidly-connected 31 wires by a single solid rod gives the same or similar results of the calculated dispersion curves. Then the dispersion curves of the whole multi-wire rope could be calculated as the curves of a bundle of solidly-connected six strands (the overall diameter 35 mm). This simplified assumption could reduce the required computational resources and additionally enable us to estimate frequency dependent leakage losses using the simplified analytical technique.

**Figure 5:**
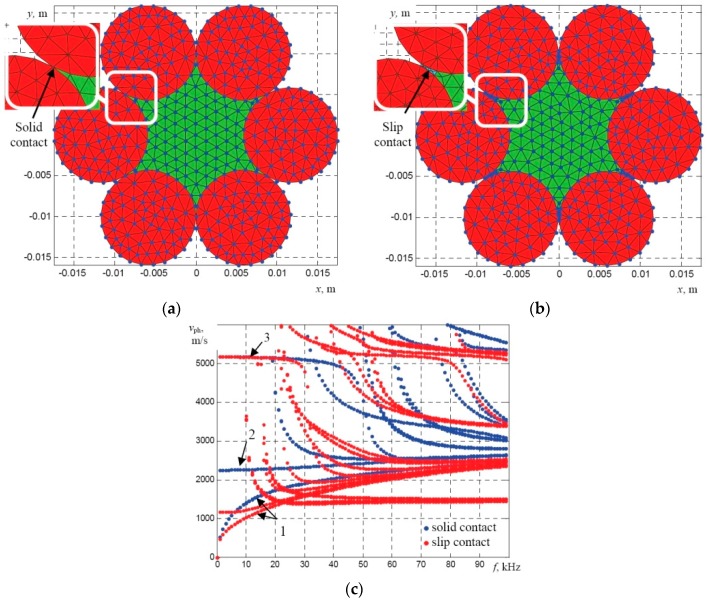
The mesh of the SAFE model of a multi-wire rope in the case of a solid (**a**) and a slip (**b**) contact between neighboring strands and comparison of the phase velocity dispersion curves of the guided wave modes (**c**) propagating along the particular structure: solid contact (**blue**), slip contact (**red**): 1—flexural-like *F*(1,1) mode; 2—torsional-like *T*(0,1) mode; 3—axial-like *L*(0,1) mode.

When conducting theoretical analysis on a multi-wire steel rope of a complex structure, an important question arises which is related to the acoustic contact between neighboring wires. Transmission of the appropriate modes between neighboring wires depends on a type of the acoustic contact. For example, in the case of a slip contact there is no transmission of the axial component of the particle velocity of the propagating mode to the neighboring wires. Therefore, only the component of the particle velocity perpendicular to the axis of the rope could be transmitted to the neighboring wires. In the case of a solid contact, the axial component is transmitted to neighboring wires. In order to investigate the influence of the acoustic contact on dispersion curves of the guided waves, the calculations for the simplified structure of the wire rope with the polymer core was performed. The calculations of the dispersion curves were performed for two separate cases. In Case A, it was assumed that the acoustic contact between neighboring wires is solid and in Case B a thin layer of a new material named as “elastic fluid” (Table 1) with the shear wave velocity close to zero was introduced between the separate strands to simulate the slip contact. The cross-section of the meshed multi-wire rope in the cases of a solid and slip contacts are presented in Figure 5a,b. The dispersion curves in the case of a solid and slip contact are presented in Figure 5c.

The results obtained demonstrate that the type of the contact between neighboring straight strands influences the type of modes that propagate in the structure. The phase velocity of the *L*(0,1)-like mode is the same for both cases at the frequencies below 30 kHz, whereas the phase velocity of the asymmetrical *F*(1,1)-like mode is shifted down in the case of the slip contact. It can be seen that in the case of the slip contact a large number of higher-order modes propagate in the structure at the frequencies above 10 kHz.

### 2.2. Investigation of the Acoustic Contact Between Wires Using 3D FE Modelling

Dispersion curves of UGW show what modes may propagate along a multi-wire rope. The most appropriate way to excite UGW in such structures is to apply a normal (perpendicular to the axis of the rope) or tangential force to the outer surface of a wire rope (parallel to the axis of the rope). It is a promising solution for practical applications, based on a single side access to the surface of the rope. Thus, the next stage of investigation must be devoted to the issue whether those UGW modes can be excited from the outer surface and whether such propagating waves penetrate into the whole structure of a wire rope or just propagate only along the outer wires. In this case, a very important task is determination of the type of the acoustic contact between neighboring wires in a rope. Each wire is attached to another one by some force and in general, two types of acoustic contact between wires should be analyzed—a solid contact and a slip one. Those contact types influence what components (perpendicular to the axis of the rope or axial) of the particle velocity can propagate from one single wire to another one and, as a consequence, determine how deeply the propagating guided wave can penetrate into the structure under investigation. In order to get the answer to these questions, two models had been constructed using the finite element method with two different types of the acoustic contact between wires—a solid and a slip contact. In the case of the solid contact vertical (*v*_y_) and axial (*v*_z_) components of particle velocity are transmitted. Still, in the case of the slip acoustic contact only components (*v*_y_) perpendicular to the rope axis are transmitted.

Elastic wave propagation can be described by the Navier equation of motion in the form of matrices [22]:

(1) $$M\ddot{U}+C\dot{U}+KU=F$$

where M is the mass matrix, C is the damping matrix, K is the stiffness matrix, F is the load vector, U is the vector of displacements, $\dot{U}$ is the vector of velocity, $\ddot{U}$ is the vector of accelerations for each degree of freedom. For the undamped forced vibrations, the Equation (1) reduces to:

(2) $$M\ddot{U}+KU=F$$

The equation above was solved using the commercially available ANSYS implicit software and the Newmark’s time integration scheme. In the case of a solid contact it was assumed that the contact layer between the adjacent wires has the properties of steel. In the case of a slip contact the layer between wires was simulated as solid, but with the shear wave velocity close to zero. In this case the shear wave is not transmitted between adjacent wires. The properties of two types of the contact layers between neighboring individual wires are presented in Table 1.

**Figure 6:**
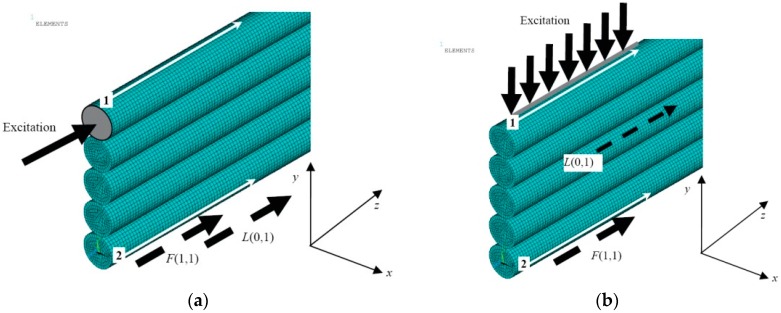
The sketch of FE model in the case of the edge type (symmetric) (**a**) and top (asymmetric) (**b**) excitation, when the diameter of a single wire is 2 mm and different types of the acoustic contact between neighboring wires was implemented: solid and slip. The numbers 1 and 2 denote the particular line for monitoring penetration of the guided wave into the structure.

Just for simplicity not the whole structure of the strand of the multi-wire rope but only a row of five wires located on each other were analyzed (Figure 6). The diameter of a single wire in the model was 2 mm. The length of the simulated structure was 250 mm. Wires were meshed using SOLID45 elements which are used for modelling of solid structures. Each element had eight nodes with three degrees of freedom each: translation in *x*, *y*, and *z* directions. The average spatial size of the elements was 0.2 mm.

For excitation of the UGW, the appropriate force for a three-period burst and the frequency of 60 kHz with the Gaussian envelope were applied in order to investigate wave propagation effects in more detail. Motivation of selection of 60 kHz frequency was based on the fact that, at a higher frequency, the wavelength of guided waves is two times shorter for the axial-like *L*(0,1) mode and one and a half times shorter for the flexural-like *F*(1,1) mode comparing to the case of 30 kHz. The interaction effects of propagating the *F*(1,1)-like mode with different types of the acoustic contact between the neighboring wires are stronger at the frequency of 60 kHz due to a lower ratio of displacements of radial and axial components. For the *L*(0,1)-like mode there is no significant difference due to a weak radial component. In addition, at mentioned both frequencies only the fundamental modes (e.g., the *L*(0,1)-like and the *F*(1,1)-like) propagate along the single thin wire and the influence of higher modes does not exists.

The symmetric mode in the upper wire was generated by applying a normal force to the edge of it covering the whole cross-section having the area of 3.3 mm^2^ (Figure 6a). The asymmetric mode was generated applying the normal force to the top surface of the same wire, the length of the excitation region was 10 mm and the area of 10.45 mm^2^ (Figure 6b). The bandwidth of the excitation pulse at −6 dB level was equal to 32 kHz. The integration step in the time domain was 0.5 μs. This step is 1/33 of the period in the case of the excitation pulse possessing the central frequency of 60 kHz.

**Figure 7:**
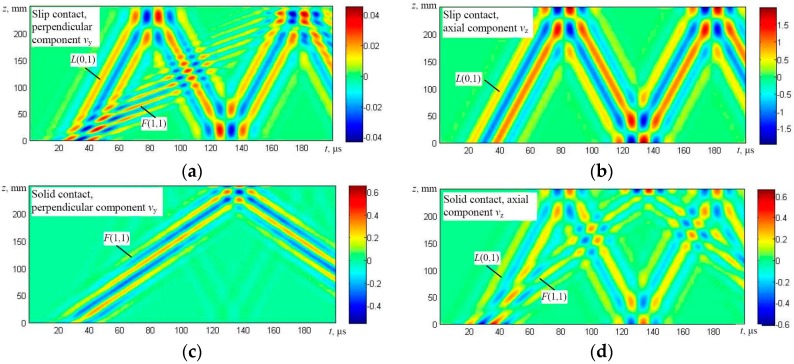
The B-scan images of the particle velocity along the first line of monitoring (the upper surface of the first wire) in the case of the edge type excitation: particle velocity components *v*_y_ perpendicular to the axis of the wire (**a**) and *v*_z_ (longitudinal-axial) (**b**) in the case of a slip contact and particle velocity components *v*_y_ perpendicular to the axis of the wire (**c**) and *v*_z_ (longitudinal-axial) (**d**) in the case of a solid contact between the neighboring wires.

**Figure 8:**
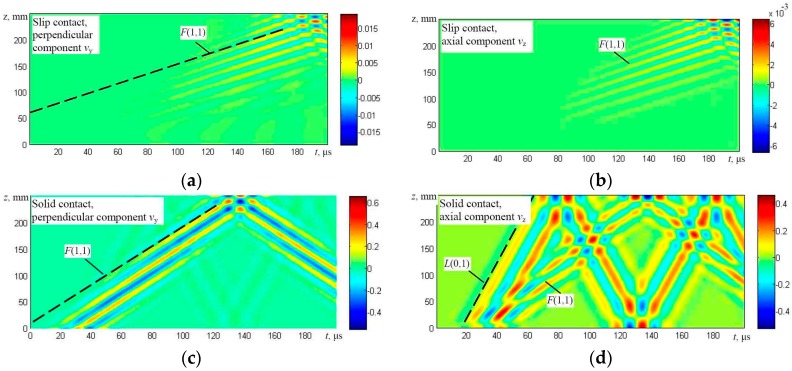
The B-scan images of the particle velocity along the second line of monitoring (the lower surface of the fifth wire) in the case of the edge type excitation: particle velocity components *v*_y_ perpendicular to the axis of the wire (**a**) and *v*_z_ (longitudinal-axial) (**b**) in the case of a slip contact and particle velocity components *v*_y_ perpendicular to the axis of the wire (**c**) and *v*_z_ (longitudinal-axial) (**d**) in the case of a solid contact between the neighboring wires.

**Figure 9:**
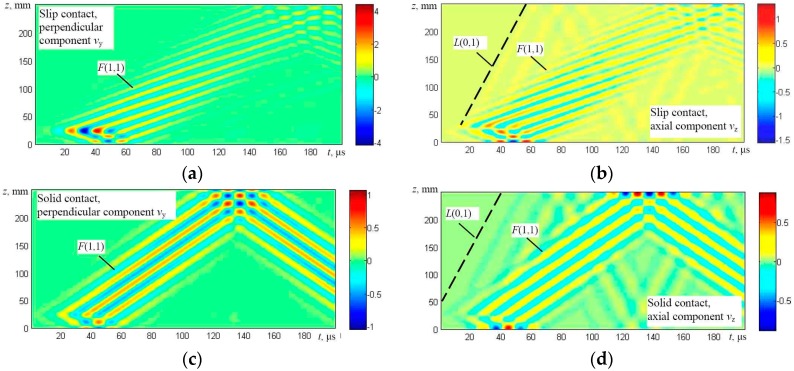
The B-scan images of the particle velocity along the first line of monitoring (the upper surface of the first wire) in the case of excitation from the top: particle velocity components *v*_y_ perpendicular to the axis of the wire (**a**) and *v*_z_ (longitudinal-axial) (**b**) in the case of slip contact and particle velocity components *v*_y_ perpendicular to the axis of the wire (**c**) and *v*_z_ (longitudinal-axial) (**d**) in the case of a solid contact between the neighboring wires.

**Figure 10:**
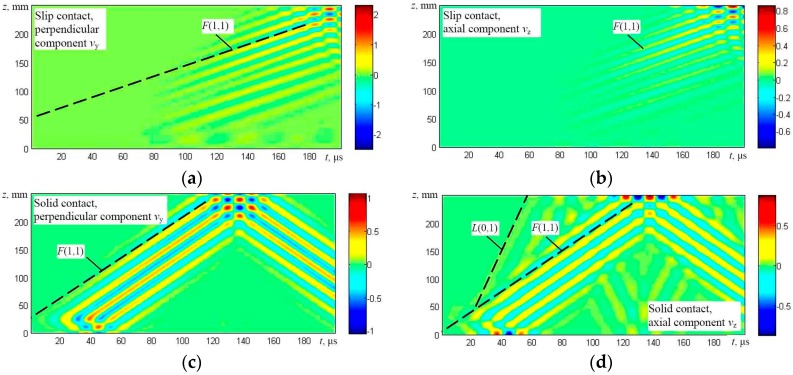
The B-scan images of the particle velocity along the second line of monitoring (the lower surface of the fifth wire) in the case of excitation from the top: particle velocity components *v*_y_ perpendicular to the axis of the wire (**a**) and *v*_z_ (longitudinal-axial) (**b**) in the case of the slip contact and the particle velocity components *v*_y_ perpendicular to the axis of the wire (**c**) and *v*_z_ (longitudinal-axial) (**d**) in the case of the solid contact between the neighboring wires.

The solution was obtained using numerical integration and in this way the particle velocity distribution in the whole simulated structure at different time instants was obtained. The results are presented in the form of B-scan images (Figure 7, Figure 8, Figure 9 and Figure 10), defining distribution of different components of a particle velocity along the central line of the upper surface of the first wire and the lower surface of the last (fifth) wire (Figure 6a,b).

It should be noted that the B-scan images have been acquired in a relatively long time interval, having a duration of 200 μs, and multiple reflections from edges of the simulated upper wire are clearly visible. The modelling results show that in the case of the edge type excitation and a slip contact between wires (Figures 7a,b and 8a,b) flexural *F*(1,1) and symmetric *L*(0,1) modes are generated in the first wire. However, only the flexural-like *F*(1,1) mode after some delay penetrates to the bottom of the structure and can be observed on the lower wires as can be seen in Figure 8a,b. In the case of a solid contact between wires and edge type excitation (Figures 7c,d and 8c,d), not only the symmetric *L*(0,1) mode, but the relatively strong asymmetric *F*(1,1) mode is generated as well, and both of them penetrate the whole structure up to the last (fifth) wire (Figure 6a).

The results obtained in the case of the top side excitation by a normal force (Figure 6b) in general demonstrate that, in both cases, the dominant flexural-like *F*(1,1) mode was generated which penetrates the whole structure (Figure 9 and Figure 10). In the case of a solid contact, the symmetric *L*(0,1) mode is simultaneously generated also.

On the basis of the simulation results, it has been found that the phase velocities of the *F*(1,1)-like mode in the cases of solid and slip contacts are different. The analysis shows that in the case of the solid contact between wires, the phase velocity of the *F*(1,1)-like mode is around 1800 m/s. If the contact (during simulation) is assumed to be slip, the phase velocity decreases approximately by half, down to 900 m/s. This can be explained by the fact that, in the case of the slip contact, even the asymmetric guided wave mode *F*(1,1)-like propagates separately in each wire.

Therefore, in the case of the slip contact, there is no possibility to generate the *F*(1,1)-like mode along the overall structure of a multi-wire rope using the excitation (normal force) from the top effectively. In the case of the solid contact, the asymmetric mode *F*(1,1)-like corresponds to the fundamental mode *F*(1,1)-like of a much thicker simulated structure compared with the case of the slip contact.

Those results coincide well with the dispersion curves obtained using the analytical technique, which shows that the phase velocity of the *F*(1,1) mode in a single wire should be around 900 m/s at the frequency of 60 kHz (Figure 2).

## 3. Finite Element Simulation of Guided Wave Propagation in 3D Wire Rope Model

The penetration of the propagating guided waves into the structure of the wire rope mainly depends on boundary conditions between the particular wires. During the finite element simulations it is usually assumed that the contact between the wires is solid to simplify the complexity of the numerical model. In this case, the surface-tangential component of the particle velocity can propagate throughout the structure of the rope. However, it is common that the multi-wire ropes are oiled to increase the flexibility and reduce the wear and abrasion. In this case, the penetration of guided waves may be quite different.

**Figure 11:**
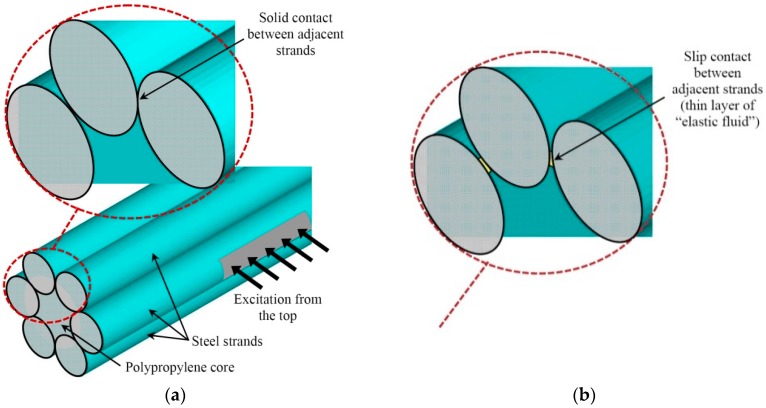
The 3D view of the multi-wire rope FE models in the case of a solid (**a**) and a slip (**b**) contact between neighboring strands.

To investigate the effects of guided wave propagation and penetration into deeper layers of the multi-wire rope, the finite element simulations of the straight 3D structure were carried out. For investigations the multi-wire rope with the polypropylene core had been selected. The length of the wire rope was equal to 0.7 m. The simulations were performed for two separate cases. In Case A, the acoustic contact between separate straight strands was assumed to be solid and had the properties of steel. In Case B, the thin layer of a new material named as “elastic fluid” with the shear wave velocity close to zero was introduced between the separate straight strands to simulate a slip contact. The 3D views of the wire rope models for the Case A and the Case B are presented in Figure 11.

The finite elements of the wire rope numerical model were created using a free mesh. The SOLID45 finite elements having eight degrees of freedom (translation in the *x*, *y*, and *z* directions) were used for meshing of the analyzed structure. The average spatial size of the elements was 2 mm for the steel region and 1.5 mm for the polypropylene. For the simulation of the layer of the “elastic fluid”, the SOLID45 elements with the average spatial size of 1 mm were used. The number of nodes per wavelength of the slowest *F*(1,1)-like mode at 30 kHz frequency was 25 for steel, 33 for the polymer, and 50 for the elastic fluid.

In both cases the guided waves were generated by applying a normal force to the top surface of a separate strand. The excitation region had the dimensions of 15 mm × 10 mm and was placed 282 mm away from the nearest edge of the wire rope. This distance is equal to a single full whorl of the particular strand around the whole untwisted multi-wire rope.

In both cases the excitation pulse had the Gaussian envelope of seven periods and the frequency of 30 kHz. The bandwidth of the excitation pulse at the −6 dB level was 4.5 kHz. The Newmark time integration scheme was applied to obtain the numerical solution. The integration step in the time domain was 1.25 µs, which is 1/27 of the period at the 30 kHz central frequency. The spatial distributions of particle velocity components (axial *v*_z_ and perpendicular to the axis of the rope *v*_x_) at different time instants were obtained using numerical integration. The C-scan images of the particle velocity distributions across the edge of the wire rope were created for the cases A and B. The C-scan images were created by selecting the peak-to-peak amplitude of the waveform at each available node of interest at time instants 55 µs, 81 µs, and 117 µs. Some further investigations demonstrated that the C-scan across the edge of the wire rope fully describes the spatial distribution of the wave amplitude.

**Figure 12:**
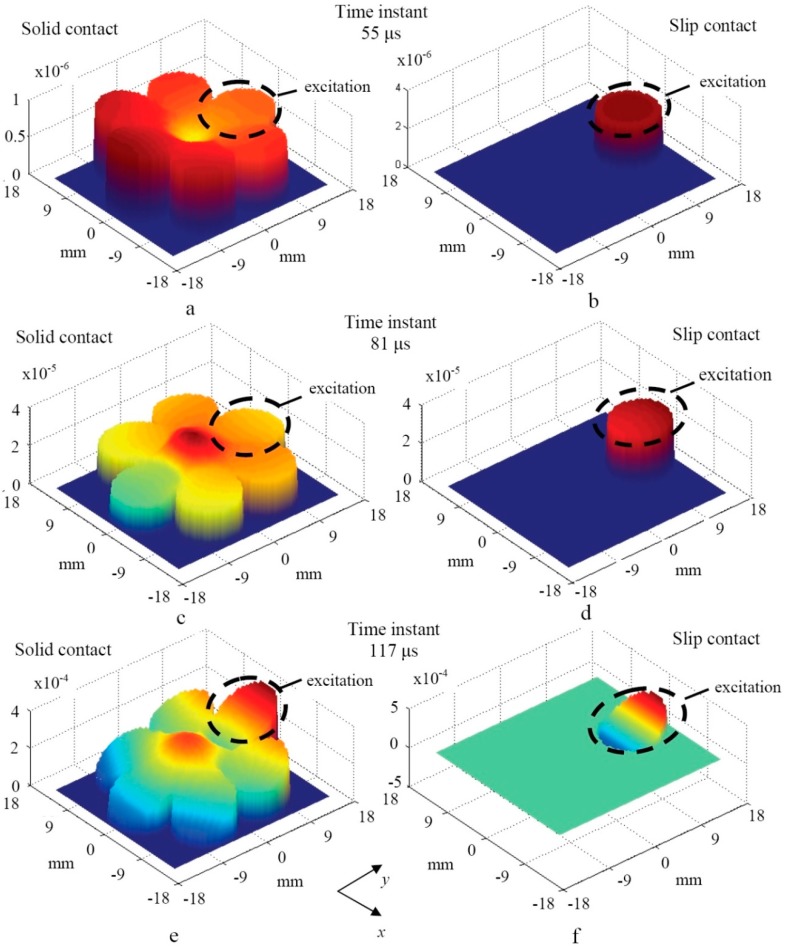
3D representation of the axial component (*v*_z_) of the particle velocity (cross-section plane *y*-*x*) at different time instants and acoustic contacts (solid or slip) between adjacent strands: time instant 55 μs ((**a**) solid contact, (**b**) slip contact), time instant 81 μs ((**c**) solid contact, (**d**) slip contact) and time instant 117 μs ((**e**) solid contact, (**f**) slip contact). The excitation was applied to the strand which is marked by a dashed line.

The C-scan images of the axial *v*_z_ component (cross-section plane *y*-*x*) of the particle velocity obtained across the selected plane in the cases of a solid (case A) and a slip (case B) contacts are presented in Figure 12a–f. Please note that the amplitude scales in the C-scan images are different at different time instants and are increasing in time. The amplitudes at the time instant 117 µs are approximately 100 times larger than at 55 µs. The axial *v*_z_ component was selected for analysis in order to verify the obtained results by relevant experiments described further.

In the case of the solid acoustic contact mainly the flexural-like mode *F*(1,1), which propagates forward, is strongly expressed within the overall cross-section of the multi-wire rope (Figure 12a,c,e). In the case of the slip acoustic contact the *F*(1,1)-like mode propagates along the single strand only (Figure 12b,d,f).

## 4. Experimental Investigation of a Multi-Wire Rope

From the numerical simulations it follows, that the penetration depth of the propagating UGW (mainly the flexural-like mode *F*(1,1)) into the internal structure of a multi-wire rope depends on the type of the acoustic contact between single wires and strands. The objective of the experimental investigation was to verify the effects of UGW propagation discovered by the FE simulation and to determine whether UGW propagate along the whole structure of wire rope or just in separate, externally-located strands.

The main purpose of the experiment was to reconstruct the wave penetration profile across the edge of the mock-up sample of the multi-wire rope. For the experimental investigation a composite rope with overall diameter of 35 mm and polymer core was selected (Figure 1).

In order to get the spatial distribution of the axial component amplitude across the edge of the multi-wire rope the air coupled technique was chosen. The immersion technique in this case is not suitable since the main goal of this experiment was to investigate the contact between separate strands. The water ingress can change the boundary conditions between the adjacent strands which would lead to inaccurate final results. The contact measurement technique was not suitable since it requires a stable acoustic force within the whole cross-section of the rope.

**Figure 13:**
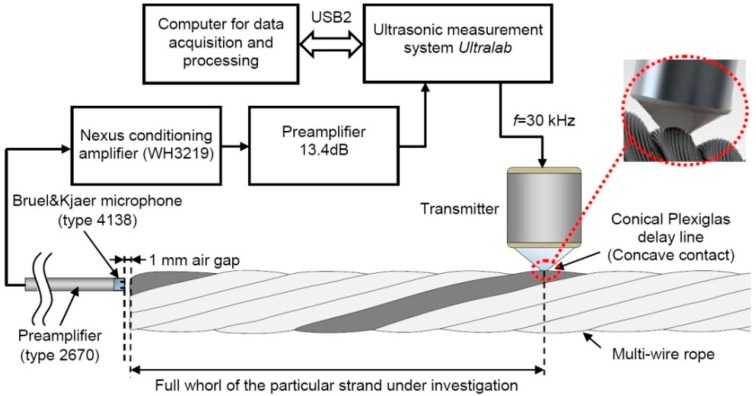
The experimental setup for the investigation of a guided wave propagation in a multi-wire rope.

The experimental investigation of the wire rope was performed using the “Ultralab” ultrasonic measurement system, which had been developed at the Ultrasound Institute of Kaunas University of Technology. The experimental set-up for investigation of the multi-wire rope is presented in Figure 13. The ultrasonic guided waves were excited using the low frequency (*f* = 30 kHz, with a bandwidth of 20 kHz at the −6 dB level) ultrasonic transducers operating in the thickness mode (in order to create the normal force) and pitch-catch configuration. The transmitting transducer was attached to the surface of the particular strand using the specially developed spring-type adjusters. The contacting area of the conical delay line of the actuator of a concave shape was 134 mm^2^ (projection of the contact area is the ellipse with the major radius of 8.6 mm and the minor radius of 5.8 mm). The transmitter was placed 272 mm away from the edge of the rope, which was equal to a single full whorl of the particular strand in the multi-wire rope. This distance is 10 mm shorter in comparison to the distance used in the finite element simulation (282 mm) as during the experiments the helical wire rope was used. To increase the transmission of the excitation force to the specimen a special conical delay lines with a concave contact surface were used. The transmitter was driven by the seven-cycle, 600 V tone burst with the central frequency of 30 kHz.

The amplitude distribution across the edge of the rope was obtained by spatial scanning of a high frequency Brüel & Kjær air-coupled microphone (type 4138, Brüel & Kjær, Nærum, Denmark, frequency range: 6.5 Hz to 140 kHz, dynamic range: 52.2–168 dB, sensitivity 1 mV/Pa). The microphone was connected to the Falcon Range preamplifier (type 2670, Brüel & Kjær, Nærum, Denmark) and the Nexus conditioning amplifier (type WH3219, Brüel & Kjær, Nærum, Denmark). The diameter of the active region of the microphone was 3.175 mm, which is close to the diameter of a single wire of the rope. Before the experiments, the edge of the rope had been polished to get a precisely flat surface. The microphone was placed 1 mm from the edge of the rope, leaving as small air gap between the receiver and the specimen as possible. The microphone was operating in the near field, as the calculated near field length, taking into account the diameter of the particular strand and the wavelength of the longitudinal mode in the air, was equal to 2.22 mm. In general, the microphone makes it possible to register the sound pressure variations in air, across the edge of the multi-wire rope.

The sound pressure can be related to the particle velocity by the equation:

(3) $$p\left(t\right)=\omega \times \xi \times Z=\frac{a\left(t\right)\times Z}{\omega }=\rho \times c\times v\left(t\right)$$

where ω is the angular frequency (rad/s), ξ is the particle displacement (m), *Z* is the acoustic impedance of air (N·s/m^3^), *a*(*t*) is the particle acceleration (m/s^2^), ρ is the density of air (kg/m^3^), *c* is the speed of sound (m/s) in air, and *v*(*t*) is the particle velocity (m/s).

**Figure 14:**
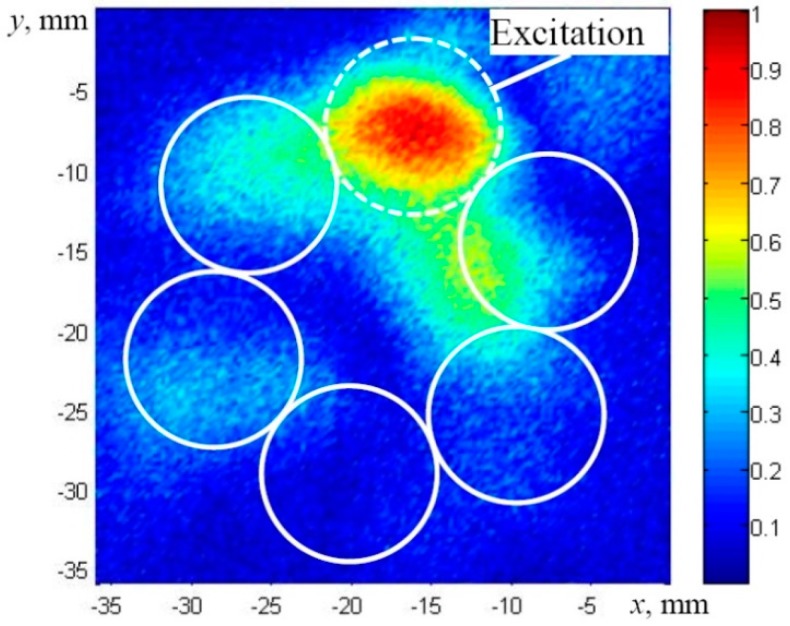
The experimentally-obtained C-scan image of the peak amplitudes of the axial components guided waves received across the cross-section of the multi-wire rope using the air coupled measurement technique. The excitation from the top was applied to the strand which is marked by a dashed line.

During the experiment, the peak-to-peak amplitudes of the axial component of the registered waveforms of the *F*(1,1)-like modes were measured within time interval of 0–250 µs. The selected relatively short time interval enabled us to distinguish between the signals propagating in the structure of the wire-rope and those which propagate directly to the microphone through the air gap. The experimentally-obtained distribution of the peak amplitudes of axial components across the tip of the multi-wire rope is presented in Figure 14. It is possible to conclude that the acoustic contact between adjacent strands of the real multi-wire rope is close to the slip type, according to the observed effect that UGW propagate mainly along the same strand on which the normal type excitation was applied. It corresponds well to the results obtained by the numerical FE simulation (Figure 12).

Previous investigations performed by contact type measurements of peak amplitudes of UGW (the *F*(1,1)-like mode) around the same strand and different strands proved that the highest amplitude of the received signal is registered when excitation and reception are performed on the same strand as well [23].

## 5. Conclusions

The important question regarding the development of the UGW-based testing technique has been the estimation of the real boundary conditions (solid or slip) between neighboring wires or strands and the real depth of penetration of UGW into the internal structure of the multi-wire rope. The propagating modes (mainly the *L*(0,1)-like and the *F*(1,1)-like) of UGW along the composite multi-wire rope have been identified by modelling using analytical, SAFE, and FE techniques. It has been estimated that some parts of the composite multi-wire rope (e.g., strands) may be replaced by a solid rod of the corresponding diameter. Otherwise, dimensions of the model are limited by a lack of computational resources and memory overflow.

During simulation of the row consisting of five wires placed on each other the obtained results have demonstrated that in the case of the edge type excitation (parallel to the axis of the wire) and the solid acoustic contact between adjacent wires, mainly the *L*(0,1)-like mode is generated. However, in the case of the solid contact, the *F*(1,1)-like mode is also generated due to mode conversions at each junction line between neighboring wires. In the case of excitation from the top by a normal force (perpendicular to the axis of the wire being excited), mainly the *F*(1,1)-like mode is generated for both types of the acoustic contact (slip and solid). The weak trace of the *L*(0,1)-like mode is also visible due to mode conversions.

The influence of different excitation regions and types of the acoustic contact between neighboring strands on the propagating UGW modes has been investigated using 3D FE modelling. Simulation results have demonstrated that in the case of the slip contact between adjacent strands and using the excitation from the top by a normal force the asymmetric-like mode *F*(1,1) propagates separately in each strand. Therefore, in the case of the slip contact the possibility to generate efficiently the *F*(1,1)-like mode along the overall structure of a multi-wire rope is limited.

From the performed numerical modelling and relevant experiments it follows that the acoustic contact between adjacent strands of the real multi-wire rope is close to slip, according to the observed effect that UGW propagate mainly along the same strand on which excitation by a normal force has been applied. Investigations have demonstrated also that even in the case of the slip contact between wires the UGW can be used for integrity analysis (e.g., non-destructive testing) of particular strands of multi-wire ropes and to detect the defective ones.

The industrial demand for this work covers application of the developed ultrasonic pitch-catch testing technique, experimental set-up and special contact-type transducers to identify the particular defective strands in a multi-wire rope.

## Figures and Tables

**Table 1:** Material properties between neighboring wires used for the calculation of the dispersion curves.

Material	Young’s Modulus	Poisson’s Ratio	Density
Steel	212.8 GPa	0.287	7800 kg/m^3^
Polypropylene core	3.54 GPa	0.36	901 kg/m^3^
“Elastic fluid”	0.075 MPa	0.499	1000 kg/m^3^
